# The C‐Type Lectin Mincle Serves as a Pattern Recognition Receptor for the Lipid Envelope of Human Cytomegalovirus

**DOI:** 10.1002/eji.70276

**Published:** 2026-09-10

**Authors:** Vladimir Bokun, Wengang Chai, Robert Buchanan, Elliot Murphy, Yan Liu, Beth Holder, Alexiane Decout

**Affiliations:** ^1^ Institute of Reproductive and Developmental Biology Department of Metabolism, Digestion and Reproduction Imperial College London London UK; ^2^ Glycosciences Laboratory Department of Metabolism, Digestion and Reproduction Imperial College London London UK; ^3^ Division of Biomedical Sciences Warwick Medical School University of Warwick Coventry UK; ^4^ Systems Medicine Department of Metabolism, Digestion and Reproduction Imperial College London London UK

**Keywords:** biology, cell biology, glycolipid, human cytomegalovirus, immune system, lectin, pattern recognition receptor, signal transduction, viral envelope, virology

## Abstract

Enveloped viruses possess a membrane composed of a complex mixture of host‐derived lipids, glycolipids, and viral glycoproteins, which play a critical role in host–virus interactions. Viral glycoproteins can be detected by a range of immune lectins, thereby modulating and sometimes subverting the host immune responses to promote immune evasion. Whether viral lipids can directly interact with pattern recognition receptors to modulate antiviral immune responses remains unclear. In this study, we report recognition of the human cytomegalovirus (HCMV) viral envelope by the C‐type lectin Mincle. Unlike previously described interactions between human lectins and viruses, Mincle recognition of HCMV and the concomitant activation of the prototypical downstream signaling pathway are mediated by host cell‐derived lipids and glycolipids incorporated into the viral envelope. Using mass spectrometry, we identify multiple previously described ligands of Mincle in the HCMV viral envelope, including cholesterol and globosides. We show that Mincle recognition of HCMV viral particles, as well as pure cholesterol, induces proinflammatory cytokine production in primary human macrophages. Collectively, these findings demonstrate that Mincle recognition of the HCMV lipid envelope constitutes a novel pattern recognition mechanism that may apply to other enveloped viruses.

## Graphical abstract

Whether viral lipids can directly interact with pattern recognition receptors to modulate antiviral immune responses remains unclear. We show that Mincle‐dependent recognition of HCMV viral particles induces proinflammatory cytokine production in primary human macrophages. These findings demonstrate that Mincle recognition of HCMV lipid envelope constitutes a novel pattern recognition mechanism.

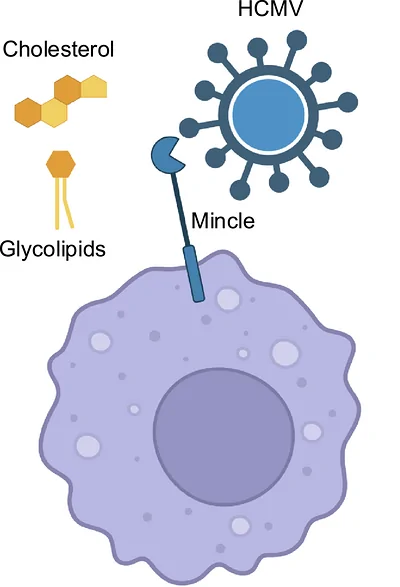

## 1 Introduction

The innate immune system is the first line of defense against pathogenic organisms and cells with abnormal function. It relies on germline‐encoded pattern recognition receptors (PRRs), which are poised to detect abnormal molecular signatures absent from the healthy self and indicative of danger [1, 2]. The antiviral innate immune response is primarily driven by the detection of cytosolic and endosomal nucleic acids, both RNA and DNA, by a range of PRRs including TLR 3, 7, 8, and 9, RIG‐I, and cGAS/STING [3, 4, 5]. Activation of these signaling cascades leads to the production of Type I interferons, which exhibit antiviral, antiproliferative, and immunomodulating properties and are considered a hallmark of antiviral responses [6]. Yet, antiviral immunity does not solely rely on nucleic acid sensing, and several other PRRs have been shown to detect, and sometimes be subverted by, viruses. The C‐type lectin family mediates the detection of carbohydrate motifs via carbohydrate recognition domains (CRDs) that typically contain one or more calcium‐dependent carbohydrate binding sites [7, 8]. Viruses derive glycans from the host cell; however, their composition is often different from that of the producing cells. For instance, high‐mannose *N*‐glycans are enriched in many viruses, and these can be recognized by several C‐type lectins [8, 9]. High‐mannose *N*‐glycans of the human cytomegalovirus (HCMV) and the human immunodeficiency virus (HIV) are recognized by dendritic cell‐specific ICAM‐3‐grabbing nonintegrin (DC‐SIGN), and this interaction can affect the immune response and promote virus internalization and dissemination [10, 11].

Apart from viral nucleic acids and glycoproteins, enveloped viruses such as HCMV carry a range of lipids and glycolipids, which are also synthesized and incorporated from host cells. Interestingly, several studies suggest that HCMV modulates the host lipid metabolism and displays a lipid composition significantly different from the host cells, with cholesterol and phospholipids being particularly enriched [12, 13]. Whether this distinct lipid profile can be detected by the host immune system as a pathogen‐associated molecular pattern (PAMP) remains unknown.

Mincle is a C‐type lectin expressed on myeloid cells, first identified as the receptor for the mycobacterial glycolipid trehalose dimycolate (TDM) [14]. Despite the presence of the characteristic EPN (Glu‐Pro‐Asn) molecular motif in the CRD of Mincle, which conveys mannose‐binding properties, current evidence indicates that its ligand repertoire comprises glycolipids and lipids, as well as proteins [15, 16, 17]. This is enabled through a unique hydrophobic groove in the Mincle CRD [18]. Cholesterol and cholesterol derivatives, which are known to be present in the viral lipid envelopes [12, 13], are recognized by Mincle, and they can activate downstream signaling pathways [16, 19, 20]. Despite this, the role of Mincle in antiviral responses has not been investigated in detail. To date, Mincle has been shown to interact with the La Crosse virus [21], to modulate the immune response to MERS‐CoV [22], and to detect cell death induced by the SARS‐CoV‐2 nucleocapsid protein [23]. However, more in‐depth investigation is necessary to better define the potential roles of Mincle in viral infections.

In this study, we report that Mincle serves as a PRR for HCMV, through recognition of viral lipid envelope components, including cholesterol. This interaction triggers the classical Mincle activation in macrophages, leading to production of inflammatory cytokines IL‐6 and TNF‐α. This represents a new, unique innate immune response mechanism triggered directly by the lipids of an enveloped virus.

## 2 Results

### 2.1 Human Mincle Interacts With HCMV

**FIGURE 1:**
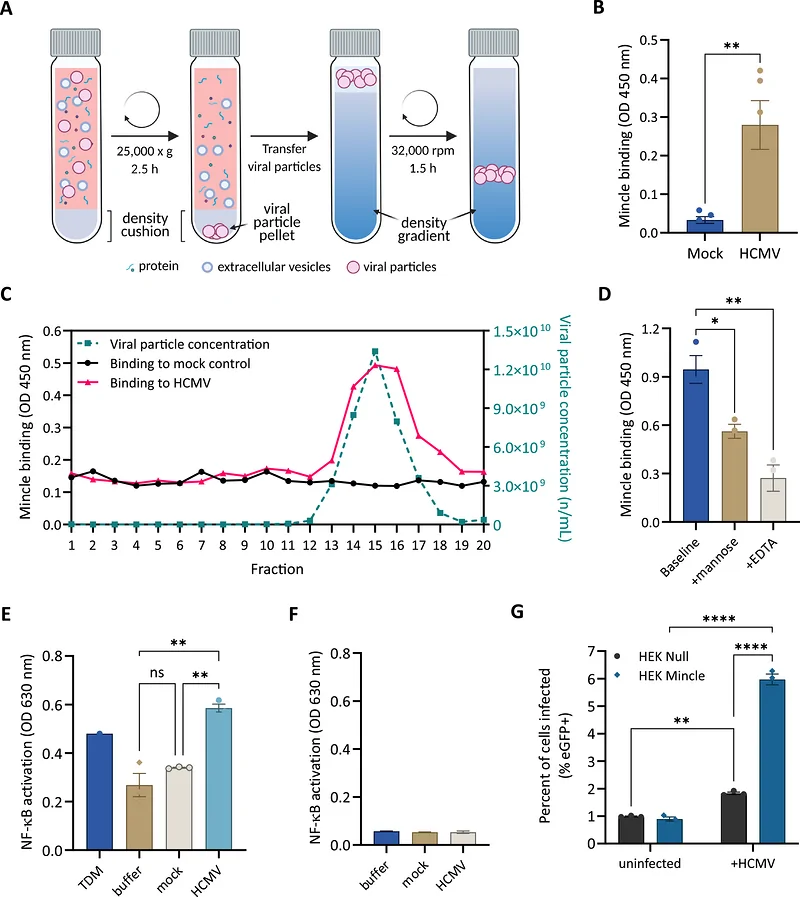
Mincle binds HCMV and activates downstream signaling. (A) Viral particles were isolated by centrifugation through density cushions and used for Mincle binding and activation experiments or further purified on a density gradient and used for Mincle binding analysis only (data in Panel C only). The density cushion‐purified HCMV viral particles were characterized by nFC and TEM (Figure S1A,B, respectively), whereas density gradient‐purified HCMV was characterized by nFC only (Figure S1C). (B) Density cushion‐purified viral particles (Steps 1 and 2 shown in Panel A) of varying titers (8.8 × 10^9^–1.6 × 10^11^ viral particles per mL), as determined by nFC, were diluted 10‐fold in DPBS and coated onto high‐binding plates. hMincle‐Fc binding was observed with the HCMV‐coated wells, but not with the mock controls prepared identically (*n* = 5 biological replicates; measured in duplicate). (C) Fractions from the density gradient purification of HCMV viral particles (Steps 3 and 4 in Panel A) were analyzed for hMincle‐Fc binding as described for Panel B. hMincle‐Fc binding was observed to fractions that contained the highest concentrations of HCMV viral particles, as determined by nFC (dotted cyan line; Figure S1C), whereas no binding was observed with the mock control fractions (*n* = 1, duplicates). (D) Inhibition of hMincle‐Fc binding to purified HCMV viral particles (titer: 1.6 × 10^11^) with 100 mM mannose and 20 mM EDTA (*n* = 3, duplicates). (E and F) Activation of the HEK hMincle cell line (E) and the matching HEK Null1 control cell line (F) by viral particles (*n* = 3, duplicates). (G) Infection of the HEK Null1 and hMincle reporter cell lines with eGFP‐expressing HCMV, assessed by flow cytometry (*n* = 4). Dot plots with gates are shown in Figure S2. Data are presented as means ± SEMs. Two‐tailed ratio *t*‐test (B); one‐way ANOVA (D and E); or two‐way ANOVA (G). eGFP, enhanced green fluorescent protein; HCMV, human cytomegalovirus; OD, optical density; TDM, trehalose dimycolate. *p < 0.05; **p < 0.01; ***p < 0.001; ****p < 0.0001.

To investigate whether Mincle recognized HCMV, viral particles were first purified by centrifugation through density cushions (Figure 1A, Steps 1 and 2), and their purity was confirmed by nano‐flow cytometry (nFC) and transmission electron microscopy (Figure S1A,B) before being probed with an Fc‐tagged recombinant human Mincle (hMincle‐Fc). This revealed interaction of hMincle‐Fc with viral particles, but not with the matching mock control from uninfected cells (Figure 1B). To further confirm that the interaction was not mediated by a contaminant from the preparations, the viral particles were further purified on a density gradient (Figure 1A, Steps 3 and 4), and each fraction probed with hMincle‐Fc. The binding signal was observed in fractions with the highest concentrations of viral particles (F14–F18), and, as before, no interaction was observed with the mock control (Figure 1C, Figure S1C). hMincle‐Fc binding to viral particles was abrogated in the presence of 20 mM EDTA and partly inhibited in the presence of 100 mM mannose (Figure 1D).

To evaluate whether Mincle binding to HCMV resulted in downstream signaling, we tested the capacity of the viral particles to induce NF‐κB activation in HEK cells expressing human Mincle (HEK‐hMincle) and an NF‐κB‐inducible reporter system (secreted alkaline phosphatase). Viral particles, but not the mock controls, indeed activated the HEK‐hMincle reporter cell line (Figure 1E), whereas no activation was observed in the parental HEK‐Null reporter cell line (Figure 1F). As Mincle has been reported to promote phagocytosis of bacteria such as *Klebsiella pneumoniae* [24], we investigated whether Mincle could impact HCMV entry into HEK cells. Flow cytometry analysis of cells infected with HCMV engineered to express enhanced green fluorescent protein (eGFP) revealed that the HEK‐hMincle cell line was more susceptible to HCMV infection than the HEK‐Null control cell line, with 6.0% of HEK‐Mincle cells and 1.8% of HEK‐Null cells infected (Figure 1G, Figure S2).

Collectively, these experiments demonstrated that Mincle was capable of recognizing HCMV and activating downstream signaling.

### 2.2 Mincle Binding and Activation by HCMV Is Mediated by the Lipid Envelope

HCMV viral envelope contains lipids and glycolipids derived from host cells. Due to the known ability of Mincle to interact with various (glyco)lipids, we hypothesized that the Mincle ligands were localized to the HCMV envelope.

**FIGURE 2:**
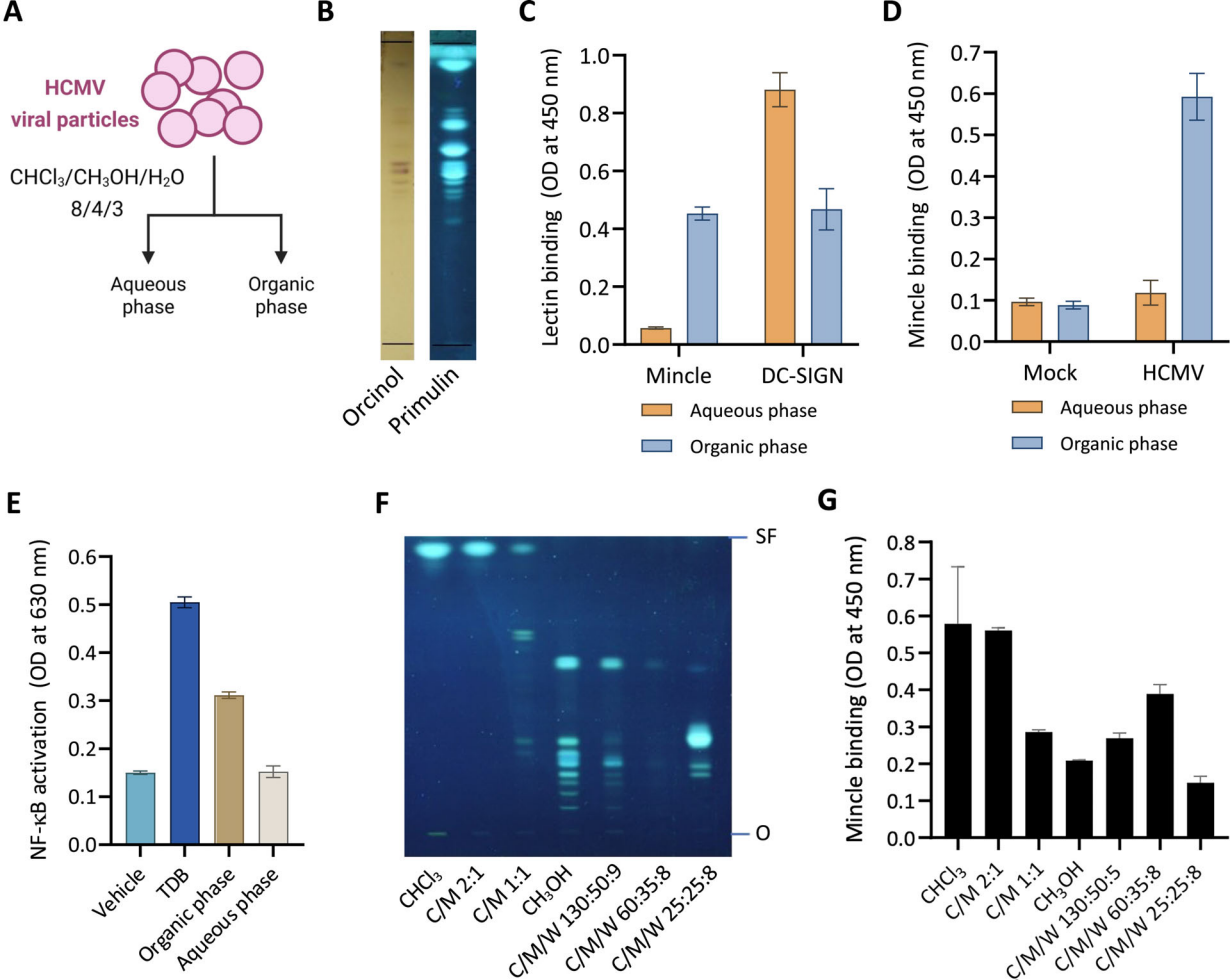
HCMV ligands of Mincle are in the lipid envelope. (A) Density cushion‐purified HCMV viral particles were solubilized and separated into organic and aqueous phases. (B) The organic and aqueous phases were analyzed by HPTLC developed with chloroform/methanol/water (60:35:8, v/v/v). The HPTLC plate was stained by orcinol (left) and primulin (right) to visualize lipids and hexose, respectively. (C) hMincle‐Fc binding was directed at the organic phase only, whereas hDC‐SIGN‐Fc bound to both phases (representative of *n* = 3, duplicates). (D) hMincle‐Fc binding was noted only to the organic phase of the HCMV viral particle extract, with no binding observed to the aqueous phase of the viral particles or either phase prepared from a mock control (*n* = 3, duplicates). (E) Activation of the HEK hMincle reporter cell line was observed with the organic phase of the HCMV viral particle extract only, along with TDB as positive control (*n* = 3, duplicates). (F) The lipid extract (organic phase) was fractionated on a C18‐silica column using a range of eluents (chloroform to chloroform–methanol–water 25:25:8). Lipid fractions were analyzed by HPTLC developed with chloroform/methanol/water (65:25:4, v/v/v). The HPTLC plate was stained by primulin to visualize the migration of the lipids/glycolipids. (G) Binding of hMincle‐Fc was observed mostly to the first two fractions (representative of *n* = 2, duplicates). Data are presented as means ± SDs. C, chloroform; HCMV, human cytomegalovirus; M, methanol; OD, optical density; TDB, trehalose dibehenate; W, water.

To examine this, we applied the Folch method [25] to extract the lipids and glycolipids from purified HCMV viral particles into the organic phase (Figure 2A). We examined the lipid extract (organic phase) by high‐performance thin layer chromatography (HPTLC), followed by primulin and orcinol staining. Primulin staining revealed several lipid bands, and orcinol co‐staining revealed the presence of hexose, indicating sugar moieties in some of the lipids (Figure 2B). When organic and aqueous phases were probed with hMincle‐Fc and hDC‐SIGN‐Fc, hMincle‐Fc interacted with the organic phase only, whereas DC‐SIGN bound to components in both phases (Figure 2C). No binding was detected to the mock control (Figure 2D). Consistently with these data, the organic phase, but not the aqueous phase, activated the HEK‐hMincle reporter cell line (Figure 2E).

To identify the lipid component(s) mediating Mincle binding, we further fractionated the lipid extract by reverse‐phase column chromatography, obtaining seven fractions of different polarities. Primulin staining revealed the presence of lipids of different polarities in the obtained fractions (Figure 2F). When the fractions were probed with hMincle‐Fc, binding was highest in the two most hydrophobic fractions, which were eluted with chloroform and chloroform/methanol 2:1 (Figure 2G).

### 2.3 The HCMV Viral Envelope Contains Multiple (Glyco)lipid Ligands of Mincle

Each lipid fraction was analyzed by electrospray ionization mass spectrometry (ESI–MS) to understand which components mediated Mincle binding.

**FIGURE 3:**
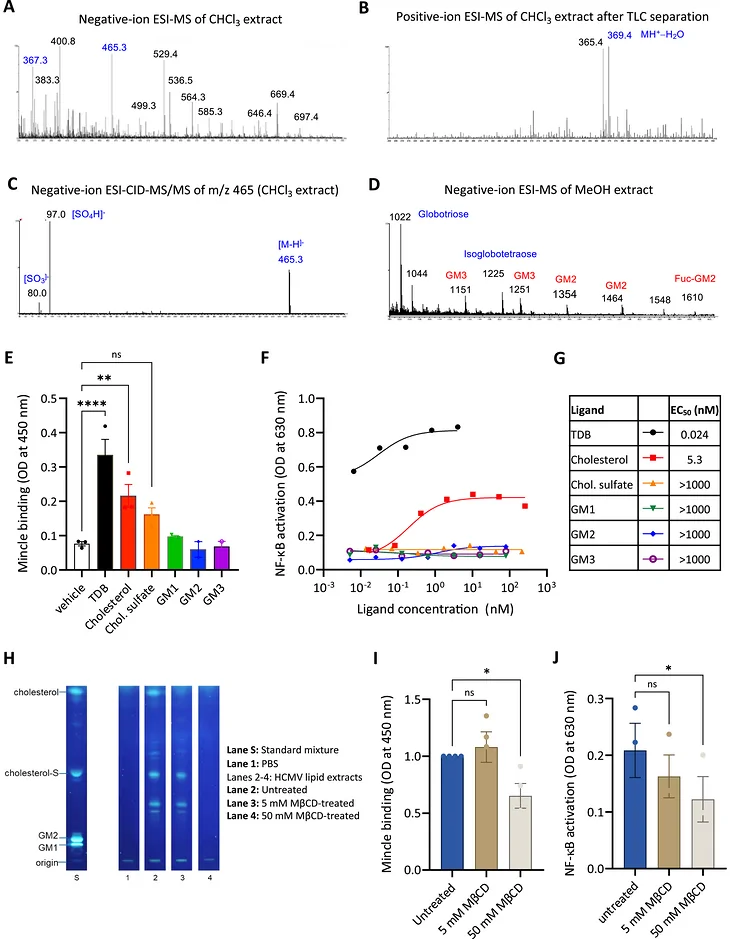
HCMV viral particles contain lipid/glycolipid ligands of Mincle, of which cholesterol activates Mincle signaling. (A) Mass spectrum of the CHCl_3_ fraction in negative‐ion detection mode. (B) Mass spectrum of the CHCl_3_ fraction in positive‐ion detection mode. (C) The product‐ion spectrum of the ion at *m/z* 465.3 (from Panel A) in negative ion mode. (D) Mass spectrum of the CH_3_OH fraction in negative‐ion mode. (E) Binding of hMincle‐Fc to pure lipids previously identified in HCMV, as well as TDB as positive control, coated at 1 µg per well (*n* = 3, triplicates). (F) Activation of the HEK hMincle cell line by pure lipids, coated at 1 µg per well (representative of *n* = 3). (G) EC_50_ values determined from the activation curves of the HEK hMincle reporter cells in panel F (*n* = 3, triplicates). (H) Lipid extracts generated from viral particles treated with 5 or 50 mM methyl‐β‐cyclodextrin were analyzed by HPTLC developed with chloroform/methanol/water (65:25:4, v/v/v) and stained with primulin to visualize lipids. (I) hMincle‐Fc binding to viral particles treated with methyl‐β‐cyclodextrin (coated at 1 µg of total protein per well), expressed as fold changes from untreated (*n* = 4, duplicates). (J) Activation of the HEK hMincle cell line by viral particles treated with methyl‐β‐cyclodextrin, coated at 1 µg of total protein per well (*n* = 3, triplicates). Additional MS data are found in Figure S3. Data are presented as means ± SDs (E) or SEMs (I and J). One‐way ANOVA (E, I, and J). *p < 0.05; **p < 0.01; ***p < 0.001; ****p < 0.0001. HCMV, human cytomegalovirus; MβCD, methyl‐β‐cyclodextrin; OD, optical density; TDB, trehalose dibehenate; TLC, thin layer chromatography.

Negative‐ion spectrum of the CHCl_3_ fraction showed many different molecular species (Figure 3A), including cholesterol ([M−H—H_2_O]^−^: 367.3) and cholesterol sulfate ([M−H]^−^: 465.3). After further enrichment, positive‐ion spectrum showed an ion at *m/z* 369.4, corresponding to cholesterol [M+H—H_2_O]^+^ (Figure 3B). The dehydrated form of the molecular ion in both negative‐ and positive‐ion spectra, with the absence of an intact molecule, was atypical. The positive‐ion spectrum of a cholesterol standard (Figure S3A) and the high‐resolution accurate mass measurement yielding an ion at *m/z* 369.3515 (theoretical *m/z* 369.3521, C_27_H_45_), corresponding to the dehydrated ion of cholesterol, confirmed the presence of cholesterol. Presence of cholesterol sulfate was also verified by negative‐ion ESI‐CID‐MS/MS (Figure 3C). This was corroborated by the spectrum of a standard (Figure S3B), which was consistent with the accurate mass measurement of the molecular ion ([M−H]^−^ found: 465.3056, theoretical: 465.3039) and the sulfate fragments ([SO_4_H]^−^ found: 96.9600, theoretical: 96.9596; and [SO_3_]^−^ found: 79.9554, theoretical: 79.9568) in the HCMV sample. The components in the chloroform/methanol 2:1 fraction (not shown) were very similar to those in the chloroform fraction in regard to their thin layer chromatography (TLC) staining patterns (Figure 2F) and Mincle binding intensities (Figure 2G).

The methanol fraction yielded multiple TLC bands of high intensity (Figure 2F), but with low Mincle binding activity (Figure 2G). Negative‐ion MS revealed many glycolipid components (Figure 3D), including globotriose (*m/z* 1022, Figure S3C) and isoglobotetraose (*m/z* 1225, Figure S3D), both with ceramide 536. Gangliosides GM3 and GM2, with ceramides 536 or 646, were also identified (Figure 3D and Figure S3E–H). Fucosylated GM2 with ceramide 646 was present in the fraction as well (Figure 3D), but its intensity was too low for CID‐MS/MS.

Overall, MS revealed a range of lipids and glycolipids, including those previously described as Mincle ligands, in the HCMV lipidome.

### 2.4 Both Cholesterol and Glycolipids Are Mincle‐Activating Ligands in the HCMV Lipid Envelope

To evaluate the relative contribution of the viral lipids to the Mincle‐mediated activation of NF‐κB signaling, we tested purified ligands for Mincle binding and activation, as done before for HCMV.

Although binding to GM1, GM2, and GM3 was not detected, hMincle‐Fc bound to cholesterol and cholesterol sulfate, though the latter was not statistically significant (Figure 3E). Consistently with the binding results, cholesterol activated the HEK‐hMincle reporter cell line in a dose‐dependent manner (EC_50_ = 3.6 nM) (Figure 3F,G). Notably, the maximum activation (max OD_630_ ∼ 0.4) was approximately 2‐fold lower than the response elicited with trehalose dibehenate (TDB) (max OD_630_ ∼ 0.8) (Figure 3F). No activation was observed for cholesterol sulfate at 1 µg/mL (Figure 3F,G), in agreement with previously published data indicating that the minimum activating concentration was 6 µg/mL [19]. No activation of the HEK‐hMincle reporter cell line by gangliosides GM1, GM2, or GM3 was observed, in line with hMincle‐Fc binding data (Figure 3F,G).

To assess the relevance of cholesterol in the context of whole viral particles, HCMV viral particles were treated with methyl‐β‐cyclodextrin (MβCD) to extract cholesterol from the lipid envelope [26]. Treatment with 5 mM MβCD decreased the intensity of the band on HPTLC migrating to a position similar to that of purified cholesterol, whereas treatment with 50 mM MβCD removed lipids in a less specific manner (Figure 3H). A solution of 50‐mM MβCD, but not 5 mM, decreased hMincle‐Fc binding to HCMV (Figure 3I). Similarly, MβCD treatment of viral particles reduced the activation of the HEK‐Mincle reporter cell line, though it did not ablate it (Figure 3J). This indicated that Mincle activation by HCMV was only partly mediated by cholesterol and that there were additional glycolipid ligands in the viral envelope that were involved.

### 2.5 Mincle Mediates an Inflammatory Response to HCMV in Human Primary Macrophages

**FIGURE 4:**
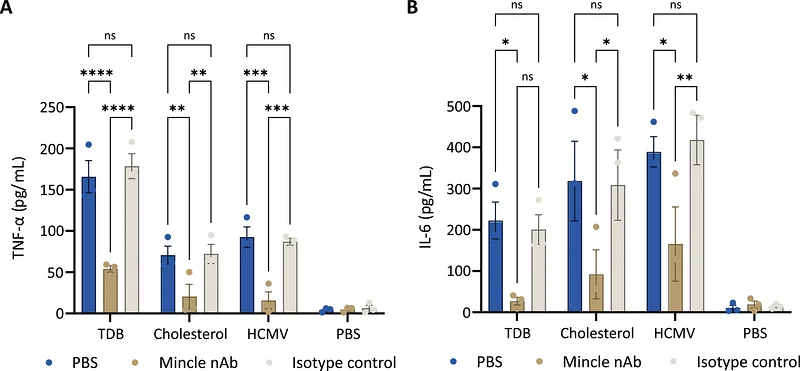
The recognition of HCMV by Mincle elicits a pro‐inflammatory immune response in primary human macrophages. (A and B) Macrophages were stimulated with 1 µg of plate‐coated lipids (TDB, cholesterol) or HCMV viral particles for 48 h, and TNF‐α (A) and IL‐6 (B) concentrations in the culture supernatant were determined by ELISA. Mincle dependence was investigated by preincubating cells with 5 µg/mL of a neutralizing anti‐hMincle antibody or a matching isotype control antibody for 30 min at 37°C before stimulation (*n* = 3, triplicates). Data are presented as mean ± SD (A and B). Two‐way ANOVA. *p < 0.05; **p < 0.01; ***p < 0.001; ****p < 0.0001. HCMV, human cytomegalovirus; TDB, trehalose dibehenate.

Mincle is expressed by several immune cell types, namely, macrophages, dendritic cells, and neutrophils [27, 28, 29, 30]. We investigated whether Mincle binding to HCMV triggered downstream signaling leading to inflammatory cytokine production in human monocyte‐derived macrophages (MDMs). TDM, cholesterol, and HCMV viral particles all activated MDMs, as evidenced by the production of cytokines TNF‐α and IL‐6 (Figure 4A,B). The activation was almost completely Mincle‐dependent, as incubating MDMs with a Mincle‐neutralizing antibody abrogated the production of the two cytokines (Figure 4A,B). No activation was observed with the PBS control, in the presence or absence of the neutralizing or isotype control antibodies.

## 3 Discussion

This study provides the first evidence of the direct recognition of a viral lipid envelope by the C‐type lectin Mincle. We identify several known ligands of Mincle in the envelope of HCMV, including cholesterol and several glycolipids. The recognition of the viral envelope by Mincle leads to the production of inflammatory cytokines IL‐6 and TNF‐α in human primary macrophages, indicating that this interaction constitutes a novel pattern recognition mechanism that actively contributes to the innate immune response.

Mincle binding to HCMV was only partially inhibited by mannose, suggesting the presence of both glycan‐based and cholesterol ligands. The binding was completely abrogated in the presence of EDTA, consistent with previous reports indicating that calcium is not only involved in the primary carbohydrate binding site, but also in the folding of the cholesterol binding site [31, 32]. Mass spectrometry analysis of the HCMV lipid extracts revealed the presence of a broad range of lipids and glycolipids, including previously identified ligands of Mincle. To our knowledge, this is the first study to employ a lipidomic analysis in the context of interactions between pathogenic viruses and immune C‐type lectins. Of all identified lipids, cholesterol was bound by Mincle, as previously described [16, 19], and it activated the HEK‐Mincle reporter cells. The absence of activation observed for cholesterol sulfate was in agreement with previous data describing a minimal activating concentration higher than the one tested in this study. In addition to these, gangliosides, previously described as ligands of Mincle [33], were also identified in the viral particles. However, none of them (GM1, GM2, or GM3) were bound by hMincle‐Fc or activated the reporter cells. This discrepancy may reflect differences in the methodologies used to establish gangliosides as ligands of Mincle, our approach being less sensitive than the label‐ and wash‐free surface plasmon resonance method employed in the previous study. The modes of multivalent presentation of the gangliosides, that is, on viral particles and plate‐coated, could also contribute to the discrepancies observed with the purified ligands. Our study focused on HCMV, which possesses a complex lipid envelope. Interestingly, enveloped viruses La Crosse virus and MERS‐CoV have previously been described to be bound by Mincle [21, 22]. However, the studies highlighting these interactions did not investigate the mechanisms of binding by Mincle, and lipidomic data are not available for these viruses. Nevertheless, it is possible that their recognition by Mincle could occur through the same mechanism as described in our work. In addition to these, SARS‐CoV‐2 has also been described as capable of activating Mincle signaling, but through an indirect mechanism involving cell death [23]. Despite SARS‐CoV‐2 being an enveloped virus, a recent lipidomic study [34] indicated that the SARS‐CoV‐2 envelope contained very low levels of cholesterol, unlike HCMV, which was described to be enriched in cholesterol [35]. This may explain why no direct activation of Mincle was observed with SARS‐CoV‐2 particles, but rather through secondary activation triggered by the nucleocapsid protein‐induced cell death [23]. Overall, two main parameters could influence the interaction of enveloped viruses with Mincle: The relative abundance and composition of lipids incorporated into the viral envelope and the accessibility of the lipid bilayer. Indeed, viral envelopes can be coated with a dense layer of viral proteins and glycoproteins that could mask immunomodulating ligands.

As the lipid envelope of HCMV viral particles contains host‐derived lipids and glycolipids, including cholesterol and cholesterol sulfate, it might be surprising that viral particles, but not the host cells, activate Mincle via their lipids. Although the lipid content of HCMV‐infected and non‐infected cells shows only minor differences, lipidomic analyses indicate that the lipid proportions of the viral envelope diverge significantly from the host cells, with cholesterol and phospholipids being particularly enriched [12, 13]. We observed that MβCD‐treated HCMV viral particles exhibited a partially reduced binding to Mincle, possibly due to viral envelope remodeling following cholesterol extraction. HCMV is known to interfere with lipid metabolism in the host cells, increasing the flux of carbon from glucose into fatty acids and cholesterol efflux. Yet, remodeling of the lipid synthesis pathways only weakly impacts the overall host cell composition. Furthermore, the viral envelope is assembled in a specialized viral assembly compartment, whose lipid composition differs from the cell surface [36]. On the basis of these findings, as well as our own, we postulate that the differential abundance of lipids, particularly cholesterol, in the HCMV viral particles might be the key characteristic that elicits recognition by Mincle, as opposed to the host cells. This hypothesis is in line with the previous report of Mincle‐mediated activation by cholesterol crystals [16] and the known requirement for clustering of lectin receptors for effective binding and downstream signaling [8, 16]. However, additional investigation is necessary to ascertain this.

Our observation that lipids found in the viral envelope of HCMV mediate the interaction with Mincle indicates that these lipids are accessible and could potentially interact with other innate immune receptors. For example, the gangliosides identified here are known to be recognized by siglecs and galectins [37, 38, 39]. Siglecs 9 and 10 have been shown to interact with gangliosides on cancer cells, activating immunosuppressive pathways [40, 41, 42]. This raises the question of whether HCMV glycolipids, or those of other enveloped viruses, could directly contribute to immune evasion mechanisms, or innate immunity, via Mincle or other glycan‐binding receptors.

Overall, our results demonstrate that Mincle is capable of recognizing the HCMV lipid envelope as a unique pattern recognition mechanism, unveiling an immunomodulatory role of the HCMV viral envelope. These findings merit further investigation of the potential interactions between Mincle and other enveloped viruses and should stimulate new research into the compositions of virus‐incorporated lipids and their contributions to innate immune response mechanisms.

## 4 Materials and Methods

### 4.1 Virus production

Human cytomegalovirus (HCMV) Merlin strain was used throughout the study [43]. R1111 strain was used for most experiments, whereas strain R1158, which contains an eGFP reporter downstream of the UL122 gene, was used for the HEK infection assay. The virus was grown in human foreskin fibroblast (HFF) cells, which were cultured in Dulbecco's Modified Eagle Medium (DMEM) with high glucose and sodium pyruvate (Sigma‐Aldrich), supplemented with 10% fetal bovine serum (FBS; from Gibco) and a mixture of l‐glutamine, streptomycin, and penicillin (to 1×; from Gibco). Virus was grown by infecting near‐confluent HFF monolayers with HCMV at a multiplicity of infection of 0.01 for 1 h at 37°C, and then replacing the virus stock with fresh complete medium supplemented with exosome‐depleted FBS (from Gibco) in place of the conventional FBS to prevent contamination with vesicles and lipoproteins from FBS. Virus was allowed to spread throughout the monolayer, and the conditioned medium was collected when all cells displayed cytopathic effects and were detaching from the growth surface (up to 10 days). The viral particles were then purified from the conditioned medium using a previously designed and characterized method [44]. Briefly, the conditioned medium was clarified by centrifugation at 300 × *g* for 5 min and 3000 × *g* for 20 min and then transferred into high‐speed centrifuge bottles (Oak‐Ridge). Overall, 16% iodixanol (OptiPrep; from Sigma‐Aldrich), diluted in DPBS (Sigma‐Aldrich), was underlaid and the bottles centrifuged at 25,000 × *g* for 2.5 h in a Beckman JS‐13.1 rotor loaded into a Beckman Avanti J25 high‐speed centrifuge at 4°C, with slow acceleration and no deceleration. The pellet, containing viral particles (virions and dense bodies), was then allowed to soak in DPBS overnight at 4°C before being resuspended and saved at −80°C. Alternatively, the resuspended viral particles were further purified on a near‐continuous 10%–36% iodixanol density gradient, constructed by layering iodixanol solutions of decreasing density (diluted in DPBS) in increments of 1%. The concentrated viral particle suspension was loaded onto the preconstructed gradients and centrifuged at 32,000 rpm for 1.5 h in a Beckman SW‐32 rotor at 4°C with minimal deceleration. Twenty fractions were collected, starting from the top of the gradient.

### 4.2 Nano‐Flow Cytometry

HCMV viral particles, comprising virions and dense bodies, were analyzed as optimized before [44]. Briefly, viral particles were fixed by the addition of 16% paraformaldehyde (Pierce) to 4% final concentration for 20 min at room temperature. The samples were then diluted at least 10‐fold in DPBS containing 1× SYBR Green I (MedChemExpress). Further dilution was made as necessary to obtain an event rate of between 2000 and 12,000 events per 1‐min acquisition. The viral particles were analyzed on a nanoFCM flow nanoanalyzer by excitation at 488‐nm (at 10 mW) and measurement of emitted side scatter through a 488 ± 5 nm bandpass filter and SYBR Green I fluorescence through a 525 ± 20 nm bandpass filter. Type 1 water was used as sheath fluid, and DPBS was used as the blank and sample diluent. Following acquisition, dot plots with *X* axis set to side scatter and *Y* axis set to SYBR Green I fluorescence were set up, and gates drawn around the viral particles to determine their concentration. The concentration was determined using a concentration standard comprising silica nanospheres of known concentration (nanoFCM), which were acquired for 1 min at the same sampling pressure as the samples (1.0 kPa). To estimate the particle size, a mixture of sizing standards comprising silica nanospheres of known diameters was used (nanoFCM). Data analysis and generation of dot plots and size distributions were performed in the same software used to operate the instrument, NF Profession, v2.13.

### 4.3 Transmission Electron Microscopy

HCMV viral particles isolated by high‐speed centrifugation through iodixanol density cushions, as described previously, were deposited onto 300 mesh carbon‐copper grids (Agar Scientific) for 5 min at room temperature. The grids were previously glow‐discharged by plasma for 50 s using a modified Fischione NanoClean Model 1070. The excess liquid was carefully blotted off the grid using absorbent paper, and the grid briefly washed with Type 1 ultrapure water. The sample on the grid was then briefly washed with 2% uranyl acetate and then incubated with 2% uranyl acetate for 45 s. The uranyl acetate solution was blotted off on absorbent paper, and the sample on grid was left to dry at room temperature. The grids were transferred into an FEI Tecnai T12 Spirit for screening, with accelerating voltage of 120 kV. The sample was screened at 6500× and 21,000× magnification, with slight defocus applied.

### 4.4 Generation of the Lipid Extracts

Viral particles were added to 750 µL chloroform/methanol/water 8:4:3 (by volume) and vortexed for 1 min. The organic phase was removed and 400 µL of chloroform added. The mixture was vortexed for 1 min, and the organic phase was removed and pooled with the previous extract. The extraction was repeated twice more, and all the organic extracts were pooled together as a total lipid extract. The collected material was left to dry under N_2_ flow.

### 4.5 Fractionation of the Lipid Extract

The lipid extract (100 µg) was solubilized in 50 µL chloroform/methanol/water 25:25:8 and loaded onto a C18 mini‐column (C18 Sep‐Pak cartridge, 100 mg; Waters, Manchester, England). Lipids and glycolipids were sequentially eluted with 200 µL each of chloroform, chloroform/methanol 2:1, chloroform/methanol 1:1, methanol, chloroform/methanol/water 130:50:3, chloroform/methanol/water 60:35:8, and chloroform/methanol/water 25:25:8. The solvents were evaporated under N_2_ flux, and all the fractions were solubilized in 100 µL chloroform/methanol/water 25:25:8 for further analysis.

### 4.6 TLC Analysis

Analysis of the total lipid extract and lipid fractions was carried out on a high‐performance TLC plate (silica gel 60, 5 µm; Merck, Darmstadt, Germany), developed in chloroform/methanol/water 60:35:8 or 65:25:4. The TLC plate was stained by primulin for detection of lipid and lipid tails of glycolipids and by orcinol for hexose detection, as described before [45].

### 4.7 Mass Spectrometry Analysis

Electrospray mass spectrometry (ESI–MS) and tandem MS with collision‐induced dissociation (CID‐MS/MS) were carried out on a Synapt G2‐S instrument (Waters, Manchester, England). High‐resolution accurate mass measurement was carried out on an IQ‐X Tribrid Orbitrap mass spectrometer (ThermoFisher Scientific, Waltham, MA, USA) at a resolution of 120,000 by direct sample injection with the Vanquish autosampler. A cholesterol standard (Sigma‐Aldrich, Poole, UK) and lipid fractions were analyzed in the positive‐ion mode, whereas cholesterol sulfate and all glycolipids were analyzed in the negative‐ion mode. Nitrogen was used as desolvation and nebulizer gas. Argon was used as the collision gas, and the collision energy was adjusted between 35 and 60 V for optimal fragmentation.

### 4.8 Removal of Cholesterol From Viral Particles Using MβCD

HCMV was grown in HFF cells and purified by high‐speed centrifugation over iodixanol cushions, as described before, and resuspended in 6 mL of DPBS. The entire sample was then split into three equal parts, and 10× stocks of MβCD (50 and 500 mM) spiked into the viral particle samples, yielding 5 and 50 mM final concentrations, along with a no‐MβCD control. The viral particles were incubated with MβCD at 37°C for 30 min. Following this, the viral particles were diluted with excess DPBS (to 32 mL per condition) and re‐purified through iodixanol density cushions as before. The viral particle pellets were then resuspended and washed once more with DPBS and re‐pelleted in a microcentrifuge at 12,000 × *g* for 15 min. Viral particles were resuspended in 500 µL of DPBS and saved at −80°C.

### 4.9 Binding of Mincle‐Fc and DC‐SIGN‐Fc

High‐binding 96‐well plates (Sarstedt) were coated with either whole viral particles in DPBS (Sigma‐Aldrich) or with isolated lipids in isopropanol. The plates were blocked with PBS containing 5% BSA for 1 h. hMincle‐Fc (R&D Systems, 8995‐CL‐050) or DC‐SIGN‐Fc (R&D Systems, 161‐DC‐050) fusion proteins (1 µg/mL in PBS, 1 mM CaCl_2_, 1% BSA) were pre‐incubated for 30 min with an HRP‐conjugated goat anti‐human IgG Fc secondary antibody (1 mg/mL, ThermoFisher Scientific, A18817) and in Figure 1D with 40 mM mannose or 20 mM EDTA and were allowed to interact with the lipids or viral particles for 2 h. Wells were washed three times with PBS, and the bound fusion proteins were detected. All steps were carried out at room temperature.

### 4.10 Mincle Reporter Cell Line Experiments

The HEK‐Blue Null1 and HEK‐Blue hMincle (InvivoGen), derivatives of HEK293 cells that stably express the human Mincle gene, along with an NF‐κB‐inducible reporter system (secreted alkaline phosphatase), were maintained in DMEM containing 10% FBS, 2 mM l‐glutamine, 100 U/mL penicillin, and 100 µg/mL streptomycin (all from Sigma‐Aldrich). Lipids or viral particles were added to 96‐well plates in isopropanol for coating, followed by evaporation of the solvent. Reporter cells (5 × 10^4^/well) were added to the ligands for 16 h, after which alkaline phosphatase activity was measured by mixing 20 µL of the culture supernatant with 180 µL of Quanti‐Blue (InvivoGen) and reading absorbance at 630 nm.

### 4.11 HCMV‐eGFP Infection Assay (Flow Cytometry)

The HEK‐Blue Null1 and HEK‐Blue hMincle cell lines were infected with the R1158 Merlin strain of HCMV, which expresses an eGFP reporter in the early phase of infection [43]. Before performing experiments, the virus was first titrated on the cells by serially diluting the virus stock and infecting the cells, followed by assessment of eGFP expression after 24 h. A dilution that produced 5%–10% of infected cells was then chosen for the final experiment. To compare infectivity of the two cell lines, the cells were infected for 24 h, collected from the plates, and fixed with 4% paraformaldehyde for 20 min at room temperature. The cells were then washed twice with PBS and resuspended in PBS supplemented with 0.1% bovine serum albumin and 2 mM EDTA. 10,000 cells per condition were acquired on a Becton Dickinson FACSCalibur flow cytometer. Dot plots with *Y* scale set to FSC‐A and *X* scale set to eGFP‐A were made, and rectangular gates were drawn based on the uninfected cells in FlowJo (version 10), and the percentages of infected cells recorded.

### 4.12 Generation of Blood‐Derived Macrophages

Buffy coats were obtained from Cambridge Biosciences (MONF05‐14P) and processed under the ethics approval BSREC 140/23‐24. PBMCs were isolated from buffy coats by density gradient centrifugation on Ficoll‐Paque (Fisher Scientific, 11768538), plated in a non‐TC‐treated well plate at 2 × 10^6^ cells/mL in RPMI 1640 (with 10% FBS, 100 U/mL penicillin, 100 µg/mL streptomycin), and incubated overnight for the monocytes to adhere. The medium was replaced with the same medium supplemented with 40 ng/mL M‐CSF (Gibco, AF‐300‐25‐2UG). On Day 3, the medium was replaced with fresh M‐CSF‐containing medium. MDMs were used on Day 5.

### 4.13 Stimulation of Blood‐Derived Macrophages

Macrophages were first primed with IFN‐γ (10 ng/mL; Miltenyi Biotec) for 3 h prior to stimulation, and then collected from the non‐TC‐treated plates by washing with PBS, followed by addition of 1× nonenzymatic dissociation buffer (Sigma‐Aldrich, C5914‐100ML) for 30 min at 37°C. The cells were washed once with PBS and resuspended at 5 × 10^5^ cells/mL in complete RPMI 1640. The cells were then incubated with either 5 µg/mL of anti‐Mincle‐neutralizing antibody (clone 15H5; InvivoGen) or a matching isotype control (mIgG2a; InvivoGen). The cell suspension of 100 µL was added to each well of the ligand‐coated plates and incubated overnight at 37°C. Supernatants were collected and analyzed for IL‐6 and TNF‐α by ELISA, following manufacturer's instructions (Invitrogen).

### 4.14 Statistical Analysis

Prism (Graphpad Software, version 10.1.2) was used to perform statistical tests and generate graphs. Two‐tailed, paired ratio *t*‐test is used for data in Figure 1B; one‐way ANOVA is used for data in Figures 1D,E and 3I,J; and two‐way ANOVA is used for data in Figures 1G and 4A,B. ANOVA was followed by Tukey's and Dunnett's post hoc tests for comparisons of all conditions to each other or to a control/baseline condition, respectively. Effects were deemed statistically significant when *p *< 0.05. Levels of significance were as follows: *p *< 0.05 (*), *p *< 0.01 (**), *p *< 0.001 (***), and *p *< 0.0001 (****).

## Author Contributions

Vladimir Bokun, Wengang Chai, Yan Liu, Beth Holder, and Alexiane Decout designed research. Vladimir Bokun, Wengang Chai, Robert Buchanan, and Alexiane Decout performed research. Data processing, analysis, and interpretation were performed by Vladimir Bokun, Wengang Chai, Robert Buchanan, and Alexiane Decout. Vladimir Bokun, Wengang Chai, and Alexiane Decout wrote the first draft of the manuscript. All authors critically reviewed, read, and approved the final manuscript.

## Ethics Statement

Study protocols were approved by the Biomedical and Scientific Research Ethics Committee of the University of Warwick (BSREC 140/23‐24).

## Consent

All donors provided written informed consent.

## Conflicts of Interest

The authors declare no conflicts of interest.

## Supporting information




**Supporting File**: eji70276‐sup‐0001‐SuppMat.pdf.

## Data Availability

The data that support the findings of this study are available from the corresponding author upon reasonable request.
