# Supplementary material for: The C‐Type Lectin Mincle Serves as a Pattern Recognition Receptor for the Lipid Envelope of Human Cytomegalovirus
Source: Eur J Immunol. 2026 Sep 10;56(9):e70276. doi: 10.1002/eji.70276 (PMC13562974; doi:10.1002/eji.70276)
Supplement: Supplementary file 1 [file EJI-56-e70276-s001.pdf]

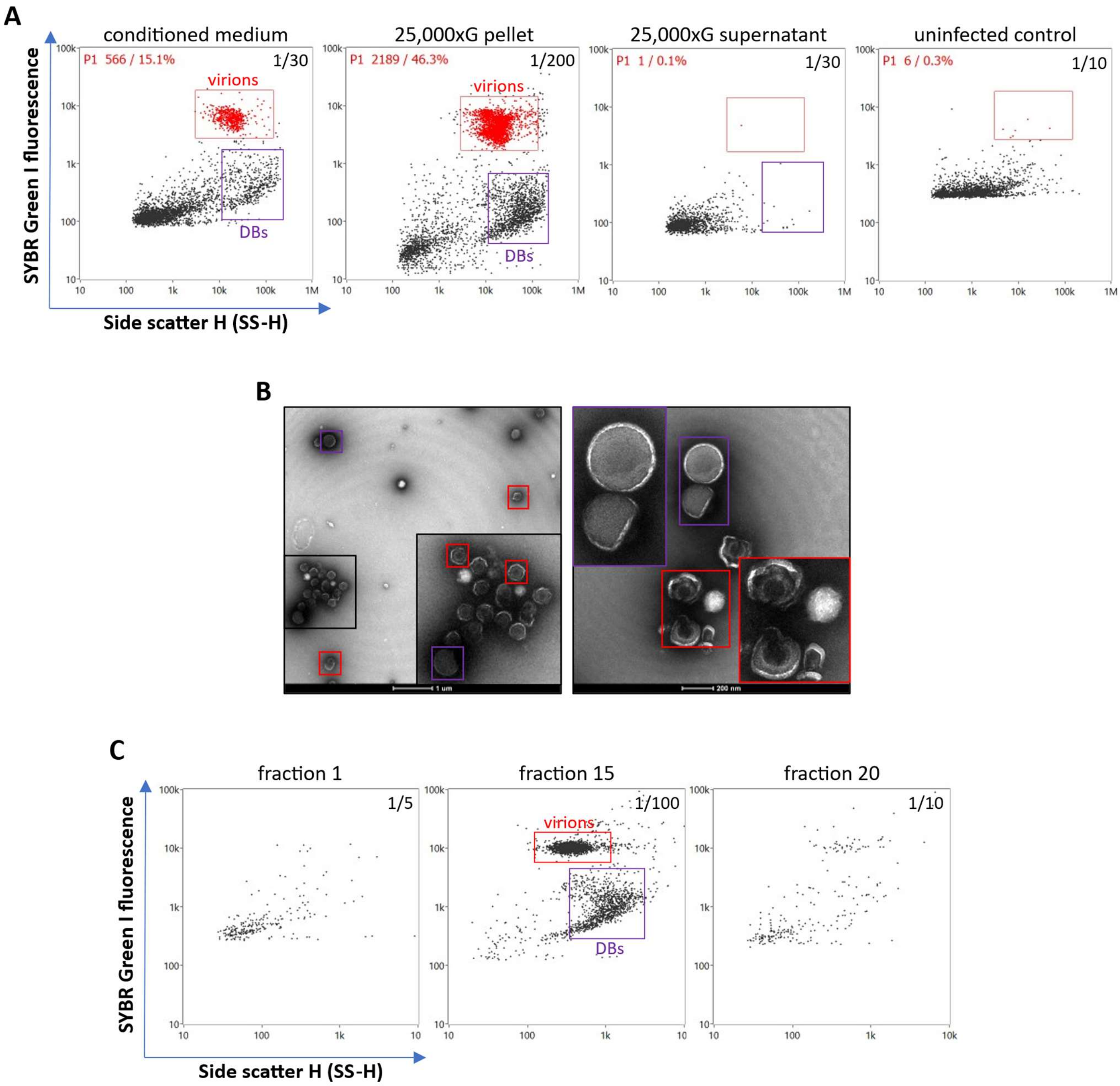

Figure S1. Characterization of HCMV viral particles by nano-flow cytometry and transmission electron microscopy. Viral particles were isolated by high-speed centrifugation through density cushions and analyzed by nano-flow cytometry (nFC) and transmission electron microscopy (TEM), as described in the 'Materials and methods' section. **(A)** Viral particles comprising virions (red gates) and dense bodies (purple gates) identified in the starting conditioned medium were significantly enriched in the isolated viral particle samples and, in turn, depleted in the centrifugation supernatants. No viral particles were observed in the uninfected (mock) controls. **(B)** The same sample was examined by TEM at 6500X (left) and 25,000X (right), revealing the presence of both viral particle types (virions = red rectangles; dense bodies = purple rectangles). No other particle types or contaminants were observed. **(C)** Viral particles were further purified on a density gradient, and fractions collected from the top to the bottom of the gradient analyzed by nFC. Viral particles were found mostly in fractions 13-17, with the highest concentration in fraction 15 (middle dot plot). Dilutions are noted in the upper right corner of each dot plot. SS-H: side scatter height; DBs: dense bodies.

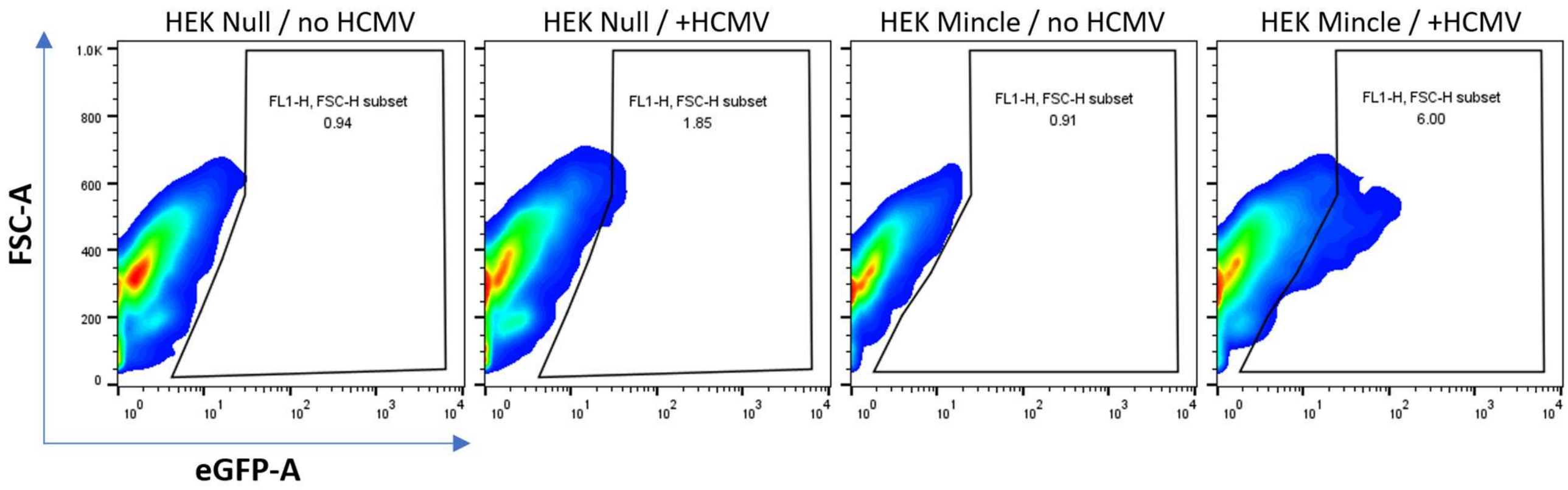

Figure S2. Infection of the HEK reporter cell lines with eGFP-expressing HCMV measured by flow cytometry. The cells were infected overnight, fixed, and evaluated for eGFP expression by flow cytometry. Rectangular gates were drawn on dot plots corresponding to infected cells. Graphs are representative of 3 independent experiments; mean values are shown in Figure 1.

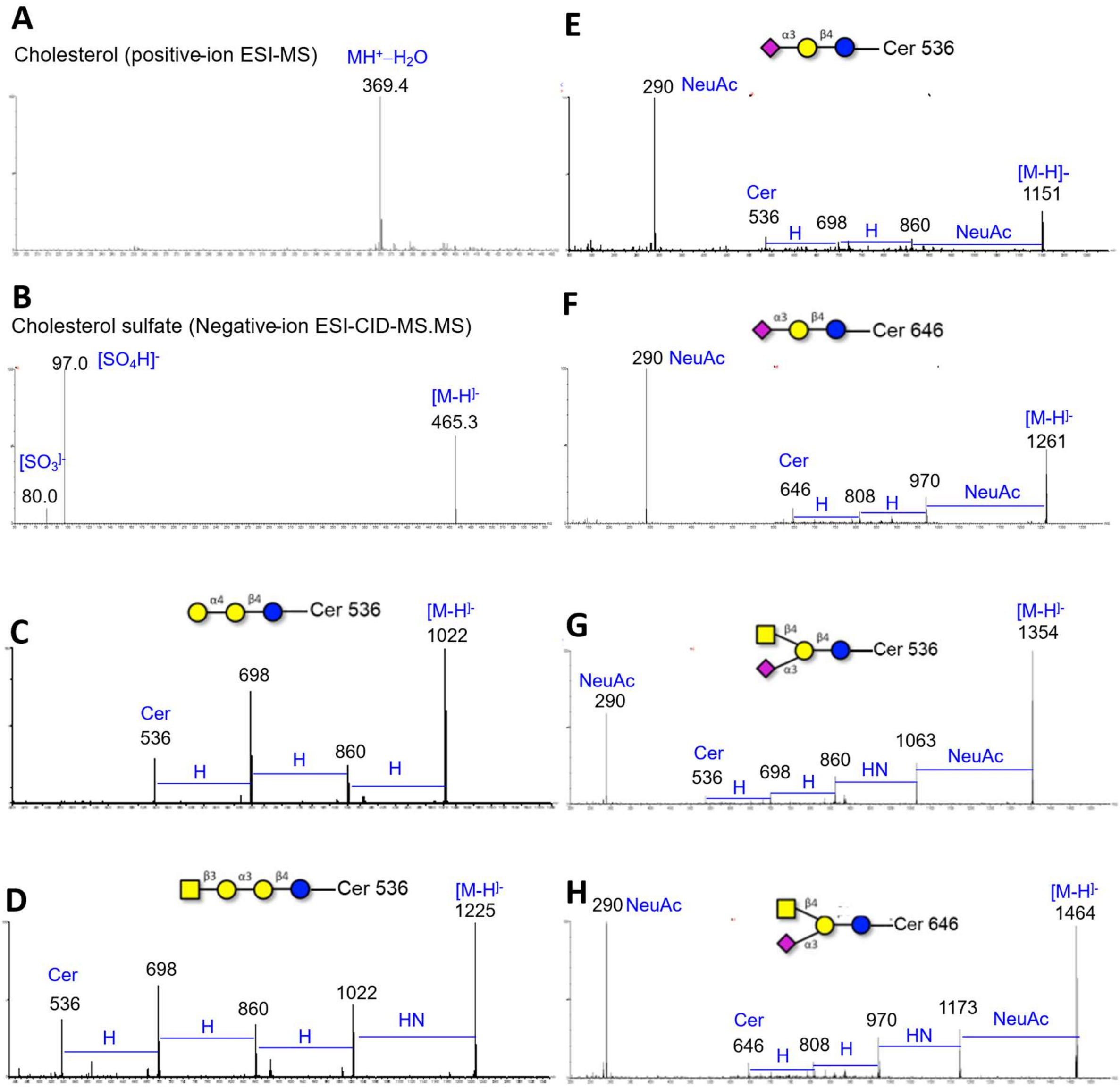

Figure S3. Mass spectrometry analysis of the lipids extracted from HCMV viral particles. (A) The mass spectrum of a cholesterol standard in positive-ion detection mode. **(B)** The mass spectrum of cholesterol sulfate standard in negative-ion detection mode. **(C-H)** The fragmentation spectrum in negative-ion mode of the CH_3OH fraction containing (C) globotriose, ion at m/z of 1022, (D) isoglobotetraose, ion at m/z of 1225, (E) GM3, ion at m/z of 1115, (F) GM3, ion at m/z of 1261, (G) GM2, ion at m/z of 1354, (H) GM2, ion at m/z of 1464.
